# Association between weight loss and mortality in idiopathic pulmonary fibrosis

**DOI:** 10.1186/s12931-022-02277-2

**Published:** 2022-12-24

**Authors:** Aleksandr Kalininskiy, Ashley Rose Rackow, David Nagel, Daniel Croft, Heather McGrane-Minton, Robert Matthew Kottmann

**Affiliations:** 1 Division of Pulmonary Disease and Critical Care Medicine, Arnot Ogden Medical Center, Elmira, NY USA; 2 Division of Pulmonary Disease and Critical Care Medicine, University of Rochester Medical Center Rochester, 601 Elmwood Ave, Box 692, Rochester, NY 14642 USA; 3 St. John Fisher College, Rochester, USA

**Keywords:** Pulmonary fibrosis, BMI, Weight loss, Mortality

## Abstract

**Rationale:**

Idiopathic pulmonary fibrosis (IPF) is a chronic, progressive interstitial lung disease that has no cure. Many current research efforts center on diagnostic and therapeutic modalities for IPF while other risk factors affecting disease pathogenesis receive less attention. Emerging data support the clinical importance of weight loss in patients with IPF. However, factors associated with weight loss and the impact of weight loss on mortality remain incompletely explored.

**Objectives:**

Explore the association between weight loss and transplant-free survival in patients with IPF and identify clinical variables associated with weight loss in this population.

**Methods:**

Kaplan–Meier and Cox proportional hazard regression analyses were generated and stratified by weight loss or use of antifibrotic medications. Conditional logistic regression was used to evaluate for factors associated with weight loss.

**Results:**

There was a significant increase in mortality in patients who lost ≥ 5% of their body weight loss (HR 2.21, [1.29, 4.43] p = .021). The use of supplemental oxygen (adjusted OR 13.16), and ≥ 200 mL loss of FVC over 1 year (adjusted OR 5.44) were both associated with a ≥ 5% weight loss in the year following a diagnosis of IPF. The use of antifibrotic medication did not significantly change median transplant-free survival in patients who lost more than ≥ 5% of their body mass.

**Conclusions:**

Weight loss over the first year following a diagnosis of IPF is strongly associated with decreased transplant-free survival. More research is needed to determine the mechanisms surrounding weight loss in patients with IPF.

## Introduction

Idiopathic pulmonary fibrosis (IPF) is a chronic progressive idiopathic interstitial lung disease (ILD) and is the most common ILD affecting older adults in the United States [1]. The reported prevalence of IPF is as high as 494 cases per 100,000 adults over the age of 65 and appears to be increasing [2]. IPF is associated with progressive lung scarring and pulmonary function deterioration, ultimately resulting in death. While the only curative therapy is lung transplant, this option is available to relatively few patients. Anti-fibrotic therapies, pirfenidone and nintedanib, reduce the rate of lung function decline [3–5] and may prolong survival but unfortunately they do not cure fibrosis [6].

Many current research efforts center on diagnostic and therapeutic modalities for IPF while other risk factors affecting disease pathogenesis receive less attention. Lifestyle factors such as dietary intake and nutritional status remain severely understudied in chronic respiratory diseases. Poor nutritional status and weight loss have been identified as independent predictors of poor clinical outcomes in chronic lung diseases such as chronic obstructive pulmonary disease (COPD) [7, 8]. Other factors including depression may also contribute to weight loss in chronic lung disease [9]. More recently, weight loss, decline in muscle mass, and decreased/low body mass index (BMI) were also associated with increased mortality in chronic obstructive pulmonary disease (COPD) [10, 11], lung cancer [12–14], and patients with IPF [15, 16]. This is clinically relevant as dietary supplementation in malnourished patients with COPD improved 6-min walking distance (6MWD), respiratory muscle strength (maximal inspiratory and expiratory pressures), and overall health-related quality of life as measured by the St. George’s Respiratory Questionnaire [17].

Emerging data support the clinical importance of weight loss in patients with IPF. A recent cohort of IPF patients from Japan and the United Kingdom showed that significant body weight (BW) decline, defined as > 6.1% body mass loss, was an independent predictor of mortality [15]. Pugashetti et al. found that a BMI decline of > 5% was associated with two times higher risk of death in patients with IPF and undifferentiated ILD [18]. Most recently Comes et al. also showed an association between weight loss and mortality in study of combined IPF, connective tissue diseases, fibrotic hypersensitivity pneumonitis and other unclassifiable types of lung fibrosis [19]. Furthermore, multiple studies have suggested that a decline in BMI may have utility as a prognostic tool [20, 21]. These studies suggest a clinically significant relationship between disease progression and weight loss.

The use of pirfenidone or nintedanib is often associated with significant gastrointestinal side effects [3, 22, 23]. A recent study showed that 63% of patients taking pirfenidone reported anorexia and 55% reported weight loss. Anorexia and diarrhea were reported in 46% of patients taking nintedanib [24]. Studies reporting associations between weight loss and mortality were published before the use of anti-fibrotic medications became the standard of care for the treatment of IPF. As such, it remains unclear how weight loss associated with anti-fibrotic therapy affects disease progression and whether anti-fibrotic treatment is as effective in patients who experience significant weight loss.

This study explores the relationship between change in weight over the first year following the diagnosis of IPF and lung function and transplant-free survival. We hypothesize that a decline in body weight by more than 5% within the first year of being diagnosed with IPF is associated with increased mortality and a significant decline in forced vital capacity (FVC). Furthermore, we aimed to elucidate whether the use of anti-fibrotic medications has any impact on weight loss and/or mortality in our cohort.

## Materials and methods

### Study population and data collection

Using an existing ILD registry database at the University of Rochester (n = 498), we identified 75 patients with a diagnosis of IPF (based on URMC MDD consensus diagnosis as outlined in the ATS/ERS/EJRS/ALAT clinical practice guideline [25]). Written consent was obtained from all subjects. Data collection was started in 2008. 71 patients had at least 1 year of longitudinal pulmonary function test data and weight measurements. Data from 34 patients were collected prior to 2014 when pirfenidone and nintedanib were FDA approved for the treatment of IPF and these patients were therefore not taking an antifibrotic medication. Patients were excluded if they did not have a diagnosis of IPF, if longitudinal or incomplete pulmonary function testing was not available, and if their weight was not documented. Diagnoses were made based on clinical history, physical exam, and CT imaging.

The date of initial IPF diagnosis was established as accurately as possible based on clinical notes. For each patient, FVC and weight/BMI were documented at the time of diagnosis and over the years following the date of diagnosis. Lung function was measured at 3-month intervals, until present date or until they had a lung transplant, when they were lost to follow-up, or were deceased. Weight measurements were recorded at the time of each PFT. Additional weight measurements were documented if they were recorded in the medical record. The change in weight and FVC at the end of the first year after the diagnosis were calculated and were only considered to be significant if the changes were consistently documented on at least three measurements. Additional variables of interest were collected including: age, gender, medical co-morbidities, smoking status, and the use of anti-fibrotic medications. This study was approved by the University of Rochester institutional review board (#00000503).

### Statistical analysis

Chi-square analyses and t-tests were conducted to compare differences in baseline demographics. Kaplan–Meier curves were generated and stratified by weight loss or use of antifibrotic medications. Cox proportional hazard regression analyses with hazard ratios (HRs) and 95% confidence intervals (CIs) were subsequently calculated.

Logistic regression was then used to evaluate for factors associated with significant weight loss. P values < 0.05 were considered statistically significant. To clarify which variables were independently associated with weight loss, a multivariate model was generated. All statistical analyses were performed using GraphPad prism version 9.1.0 for Windows, GraphPad Software, San Diego, California USA or SAS/ACCESS® 9.4 Interface to ADABAS, SAS Institute Inc 2013, Cary, NC.

## Results

### Patient characteristics

A total of 71 patients were included in the analysis (Table 1). All patients were Caucasian, all identified as non-Hispanic, 71% were male, and the average age was 71 years (SD = 9.5 years). 70% had a history of cigarette use (Table 1). There were no malignancies identified prior to or during the observation period. 51% of our cohort were on an anti-fibrotic medication. 74% percent were on an antifibrotic medication within 6 months of the diagnosis of IPF. 62% percent were on a stable dose of an antifibrotic medication for at least 2 years. None of the patients discontinued antifibrotic medication, but seven patients required a dose reduction and seven patients changed antifibrotic medications. In all cases, the changes were made after 1 year of being on an antifibrotic medication and each patient remained on a stable dose of the new antifibrotic medication for the remainder of the observation period. There were 41 deaths and 11 lung transplants over the entire period of observation. The median duration of observation in this study was 1300 days.

**Table 1:** Patient characteristics

Characteristic	All (n = 71)
Age (years)	71.3 ± 9.5
Female Gender	21 (29%)
Caucasian	71 (100%)
Weight at diagnosis (kg)	88.6 ± 17.9
BMI at diagnosis	29.81 ± 4.94
Weight change at 1 year after diagnosis	− 2.1 ± 4.3
BMI 1 yr post	29.1 ± 4.8
Smoking (any)	50 (70%)
Antifibrotic (any)	36 (51%)
Pirfenidone	19 (27%)
Nintedanib	17 (23%)
FVC at diagnosis (L)	2.9 ± 0.8
FVC % predicted	79.2 ± 19.4
DLCO at diagnosis (mL/min/mm Hg)	11.6 ± 4.7
Reflux disease	31 (44%)
Diabetes	13 (18%)
OSA	9 (13%)
Oxygen use	22 (31%)

BMI: body mass index; DLCO: diffusion limitation for carbon monoxide; FVC: forced vital capacity; OSA: obstructive sleep apnea

**Fig. 1:**
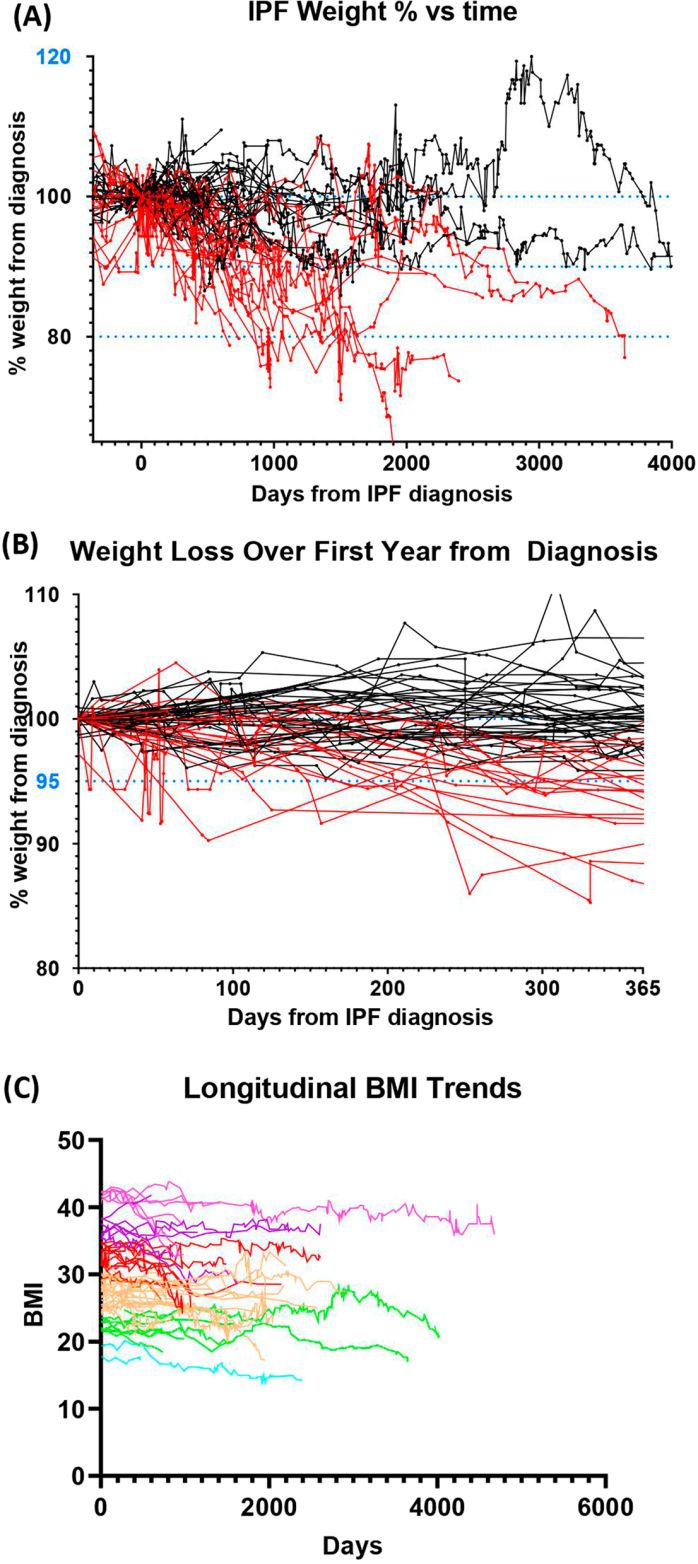
Weight-loss trajectories over time. Shown are changes in weight over the entire observation period (**A**) or stratified by those who lost ≥ 5% weight within the first year of an IPF diagnosis (**B**). Red lines indicate patients losing ≥ 5% bodyweight. Panel C demonstrates initial body mass index (BMI) at diagnosis vs. time. Colored lines on this graph represent the varying stratifications of BMI

Weight trajectories were plotted for the entire study period, and more specifically over the first year from the date of diagnosis (Fig. 1A and B). 30% of patients experienced significant weight loss over the year following the diagnosis of IPF (Fig. 1B). Only one of the patients losing more than 5% of body weight regained weight over the following 4 years. BMI trajectories were plotted from the time of diagnosis to determine whether a low BMI at the time of diagnosis was associated with greater weight loss (Fig. 1C). There were no apparent differences in weight loss on the basis of initial BMI; however, seven patients with BMIs over 30 at the time of diagnosis lost over 10% of their body mass. Similarly, when stratified by the presence or absence of weight loss within the first year, there were no significant differences in weight or BMI at the time of diagnosis BMI (Table 2). The average FVC at the time of diagnosis was 2.6 L (70.5% of predicted) in the group who lost weight and 3.1 L (82.4% of predicted) in the group who did not lose weight (p = 0.02). This suggests that patients lower FVCs may be more likely to lose weight. The group who lost weight had a higher percentage of oxygen use (57% versus 20%, p = 0.72), reflux disease (52% versus 40% p = 0.43) and utilization of pulmonary rehabilitation (24% versus 18%, p = 0.52); however, these changes were not statistically significant (Table 2).

**Table 2:** Patient stratification by the presence or absence of weight loss

Characteristic	All (n = 71)	> 5% weight loss in 1st year (n = 21)	No weight loss in 1st year (n = 50)	P-Value
Age (years)	71.3 ± 9.5	69.9 ± 8.7	71.9 ± 9.8	0.34
Female Gender	21 (29%)	7 (33%)	14 (28%)	0.78
Weight at diagnosis (kg)	88.6 ± 17.9	89.3 ± 14.1	88.3 ± 19.4	0.83
BMI at diagnosis	29.8 ± 4.94	30.6 ± 4.11	29.5 ± 5.23	0.40
Smoking (any)	50 (70%)	15 (71%)	35 (70%)	0.90
Pulmonary rehab	14 (18%)	5 (24%)	9 (16%)	0.52
Antidepressant	24 (33%)	5 (23%)	16 (32%)	0.38
Antifibrotic (any)	36 (51%)	13 (62%)	23 (46%)	0.30
Nintedanib	19 (27%)	7 (33%)	12 (24%)	0.56
Pirfenidone	17 (23%)	6 (29%)	11 (22%)	0.55
FVC at diagnosis (L)	2.9 ± 0.8	2.6 ± 0.8	3.1 ± 0.8	0.02*
FVC% Predicted	79.2 ± 19.4	70.5 ± 19.9	82.4 ± 18.3	0.02*
DLCO at diagnosis (mL/min/mm Hg)	11.6 ± 4.7	10.0 ± 4.5	12.3 ± 4.6	0.08
Reflux disease	31 (44%)	11 (52%)	20 (40%)	0.43
Diabetes	13 (18%)	5 (24%)	8 (16%)	0.50
OSA	9 (13%)	2 (10%)	7 (14%)	0.72
Oxygen use	22 (31%)	12 (57%)	10 (20%)	0.72

BMI: body mass index; DLCO: diffusion limitation for carbon monoxide; FVC: forced vital capacity; OSA: obstructive sleep apnea. *p < 0.05

### Weight loss and mortality

**Fig. 2:**
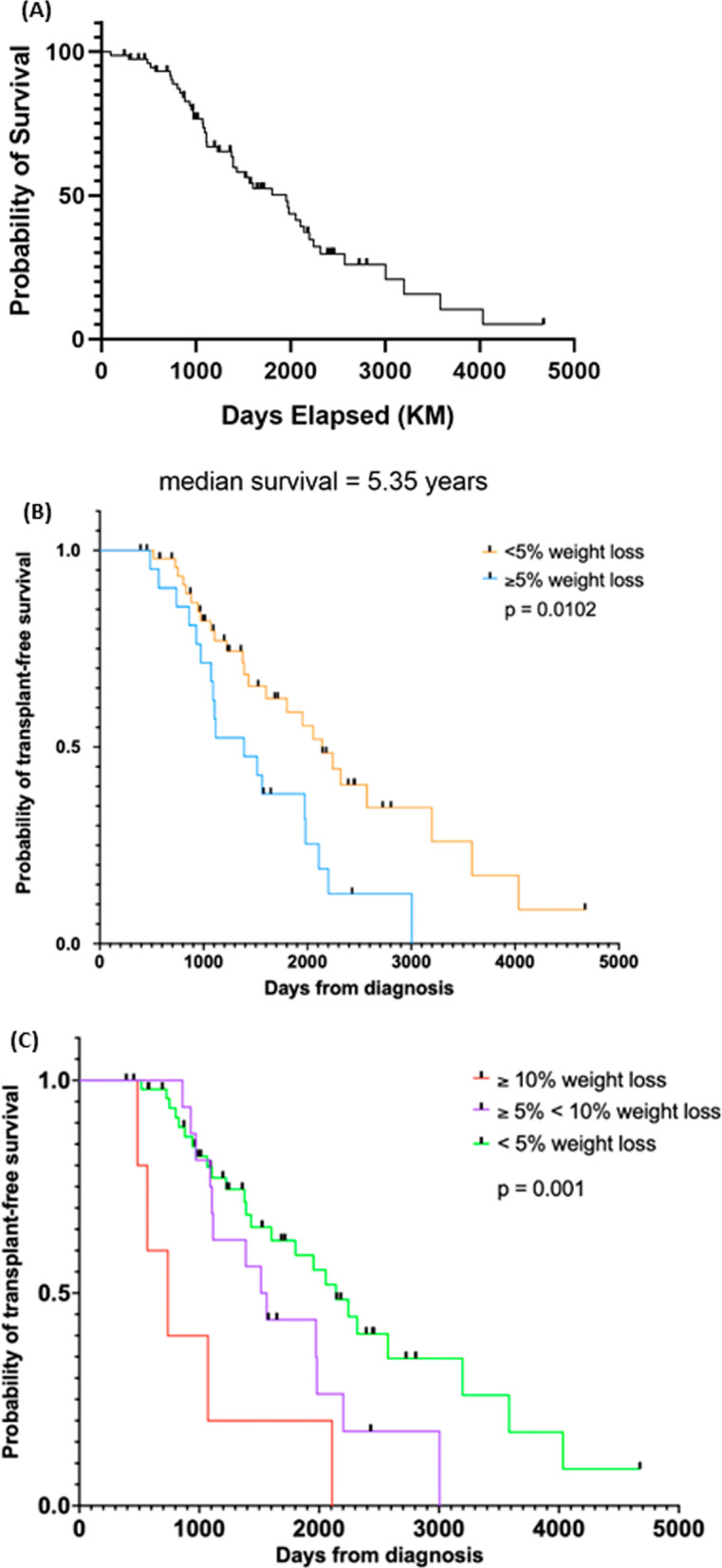
Kaplan–Meier analysis for transplant-free survival. The median survival in our cohort was 5.35 years from the time of IPF diagnosis (**A**). However, when patients were stratified by at least 5% weight loss vs those who lost < 5%. Those who lost weight had a significantly worse prognosis (median transplant-free survival of 3.8 years vs 5.8 years, p = 0.0102) (**B**). For those patients who lost more than 10% of their weight, there was an even more significant detrimental effect on transplant-free mortality (median survival of 2.02 years, p = 0.001) (**C**)

Kaplan–Meier analysis of the entire cohort showed a transplant-free median survival of 5.35 years (Fig. 2A). To examine the association between transplant-free survival and weight loss, Kaplan–Meier analyses were performed stratified by those who lost less than 5% body weight versus those who lost at least 5% body weight in the first year (Fig. 2B). The stratification clearly demonstrates there is a significant increase in mortality for those who experienced greater than 5% body weight loss (HR 2.21, [1.29, 4.43] p = 0.021). The impact of weight loss on survival remained present even after direct adjustment for age and FVC at the time of diagnosis (HR 2.03, [1.36–2.71], p = 0.038). Neither age or FVC at time of diagnosis were significantly associated with a reduction in transplant free survival in the model HRs 1.02, [0.97–1.05] (p = 0.52) and 0.87, [0.47–1.27] (p = 0.50) respectively. The median survival of patients who lost more than 5% of their body mass over the first year was 1389 days (3.8 years) while the median survival for patients who did not lose a significant amount of weight was 2142 days (5.8 years). The group of patients who lost more than 10% of the body mass (n = 4) in the first year had significantly higher mortality than the group who lost between 5–10% of their body mass, median survival 738 days (2.02 years) (Fig. 2C).

### Weight loss and FVC decline

**Fig. 3:**
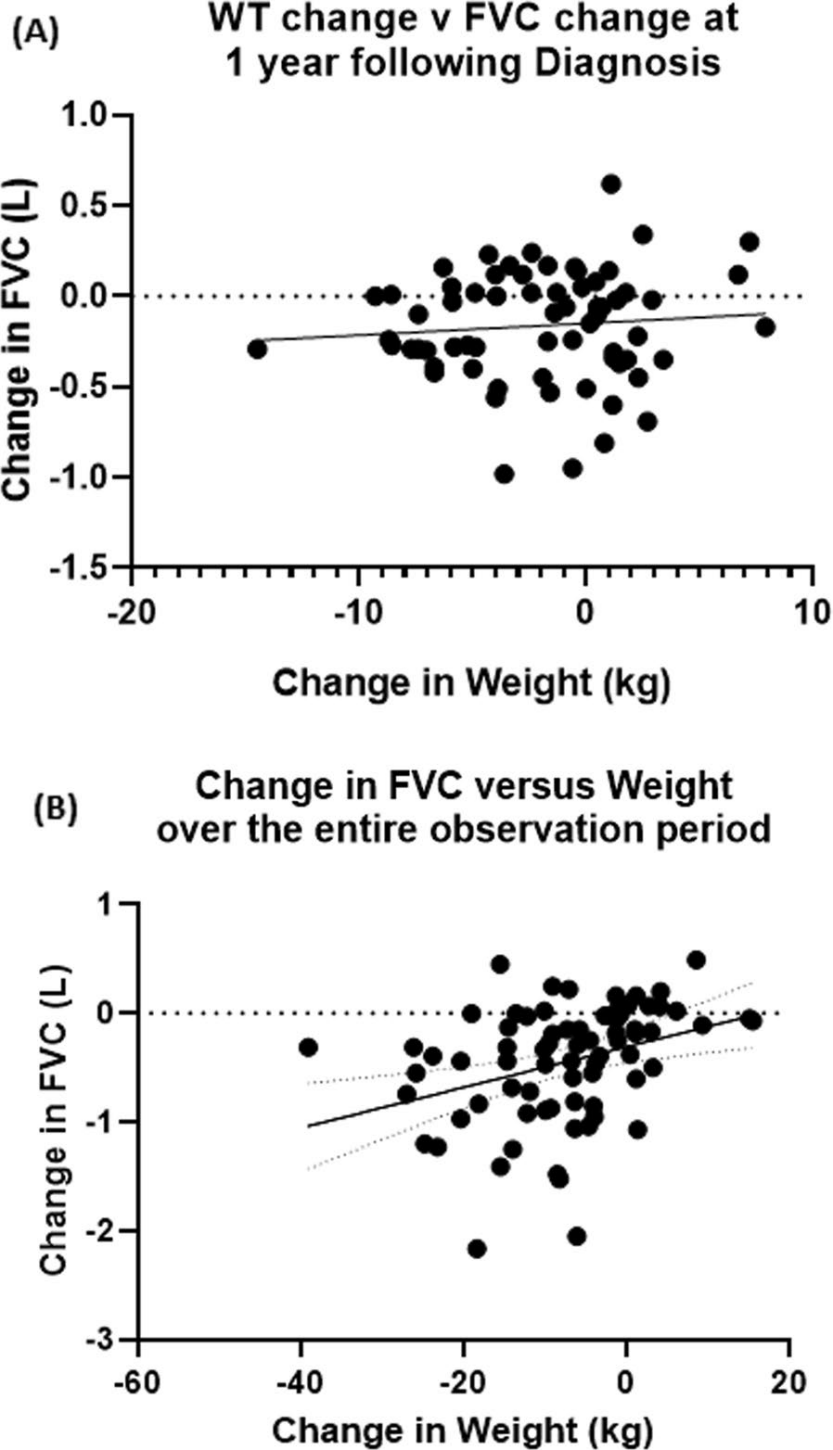
Association between weight loss and change in Forced Vital Capacity. There was no significant effect of weight change over the first year after IPF diagnosis and decline in FVC over the same time period (p = 0.46) (**A**). However, there was a significant association between change in weight vs change in FVC during the entire observation period (p = 0.0026) (**B**)

After demonstrating a clear relationship between weight loss over the first year after a diagnosis of IPF and reduced transplant-free survival, we next examined whether weight loss was associated with a decline in FVC. There was no significant linear association between weight loss and change in FVC in the year following a diagnosis of IPF (Fig. 3A) (p = 0.42). However, there was a statistically significant linear relationship between the total amount of weight lost over the observation period and the total change in FVC over the entire observation period (p = 0.002) (Fig. 3B). This does suggest that even early weight loss is highly associated with long-term lung function decline.

### Factors associated with weight loss

We next assessed whether any demographic or clinical factors may be associated with weight loss. Univariate analysis of multiple clinical variables was performed (Table 3). Several variables including anti-fibrotic use, use of an Angiotensin Receptor Blocker and gastroesophageal reflux disease (GERD) were not associated with weight loss. Although there was no clear linear association between changes in FVC in the year following the diagnosis of IPF, weight loss was highly associated with long-term lung function decline. We hypothesized that larger FVC volume losses in the first year after diagnosis may be associated with weight loss over the first year. For purposes of these logistic regression analyses, changes in FVC were stratified to large volume losses (≥ 200 mL over the course of a year) and small volume losses (< 200 mL over the course of a year). A loss of ≥ 200 mL in the first year was associated with an increased odds of weight loss (OR 3.03 [0.985, 9.341]) but this did not reach statistical significance (p = 0.053). The use of supplemental oxygen was statistically associated with weight loss (OR 5.4 [1.86–17.18] p = 0.0031). Though not statistically significant, we observed a 90% increase in the risk of weight loss in patients on antifibrotic medications in the past year when compared to patients not taking antifibrotic medications (OR 1.907, [0.673, 5.405], p = 0.22). It is important to note that this analysis did not distinguish whether either individual medication was, by itself, associated with greater weight loss.

**Table 3:** Factors associated with ≥ 5% weight loss within the first year of IPF

	OR	CI	p value
Univariate analysis			
≥ 200 mL loss of FVC	3.03	[0.985, 9.341]	0.053
Antifibrotic use	1.907	[0.673, 5.405]	0.22
ARB use	3.071	[0.917, 10.28]	0.07
ACE use	0.307	[0.035, 2.67]	0.28
Pulmonary Rehab	1.467	[0.413, 4.902]	0.57
Smoking	1.071	[0.386, 5.577]	0.90
GERD	1.65	[0.565, 4.433]	0.34
PPI	0.80	[0.286, 2.21]	0.66
COPD	0.228	[0.027, 1.924]	0.17
Diabetes	1.641	[0.467, 5.767]	0.30
Pulmonary hypertension	1.211	[0.552, 6.99]	0.83
Sleep apnea	0.647	[0.123, 3.407]	0.61
Atrial fibrillation	0.480	[0.094, 2.437]	0.38
Oxygen use	5.333	[1.762, 16.15]	0.003*
Gender	1.286	[0.429, 3.852]	0.65
Multivariable analysis			
Oxygen use	13.167	[3.059–56.68]	0.0005*
≥ 200 mL loss of FVC	5.44	[1.28, 22.98]	.021*
Age	1.034	[0.96, 1.114]	0.38

ARB: angiotensin receptor blocker; ACE: Angiotensin converting enzyme inhibitor; BMI: body mass index; COPD: chronic obstructive lung disease; DLCO: diffusion limitation for carbon monoxide; FVC: forced vital capacity; GERD: gastroesophageal reflux; OSA: obstructive sleep apnea; PPI: proton pump inhibitor. *p value < 0.05

To determine whether weight loss, ≥ 200 mL loss of FVC, and supplemental oxygen use were independently associated with a reduction in transplant free survival we generated a multivariable model using stepwise addition modelling. Additional variables included in the modelling were age, gender, and anti-fibrotic use. The final model was adjusted for age, ≥ 200 mL loss of FVC and supplemental oxygen use. The use of supplemental oxygen (adjusted OR 13.167, [3.059–56.66], p = 0.0005) and ≥ 200 mL loss of FVC over 1 year (OR 5.44, [1.28, 22.98], p = 0.02) were both associated with a ≥ 5% weight loss in the year following a diagnosis of IPF.

### Anti-fibrotic use, weight loss

**Fig. 4:**
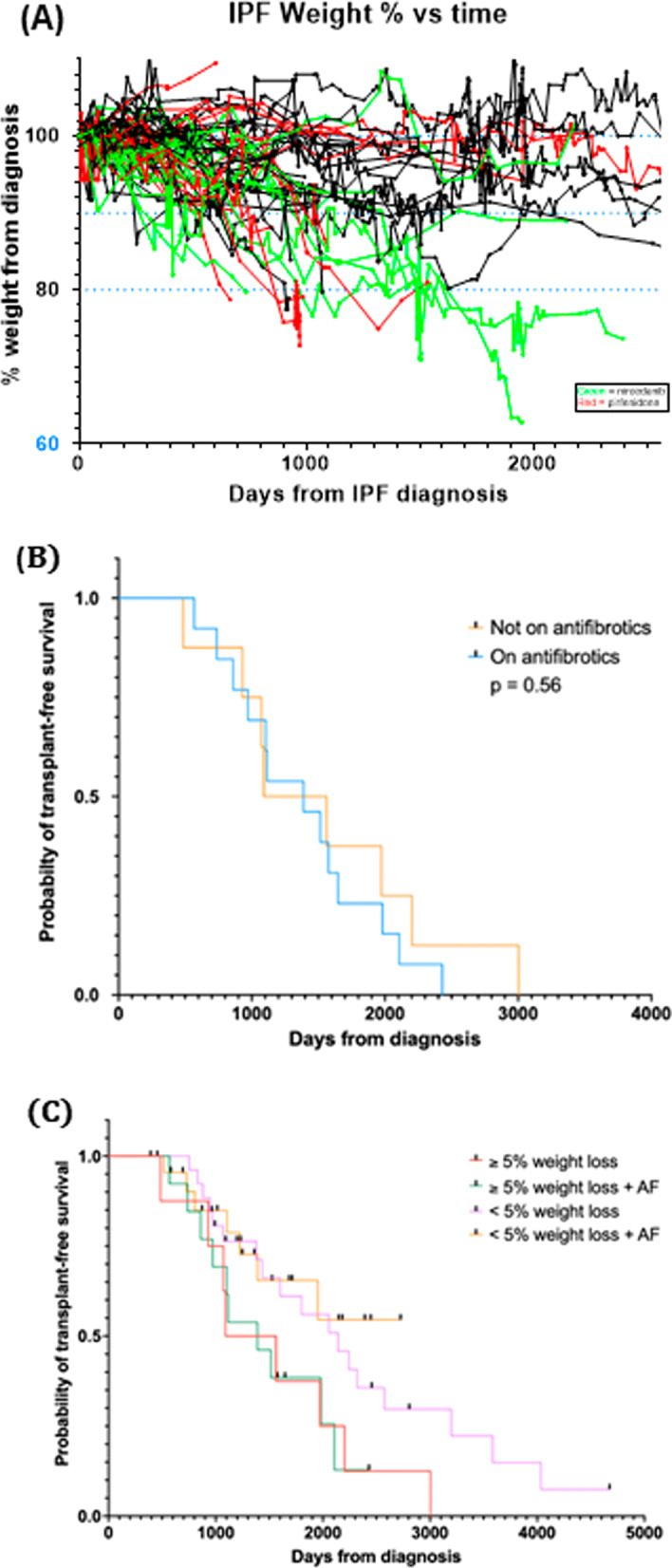
Effect of anti-fibrotic medications on weight loss and transplant-free survival. Patients taking anti-fibrotic medications demonstrated similar weight loss compared with those not taking anti-fibrotic medications. Red lines: pirfenidone, Green lines: nintedanib (**A**). There was slightly more weight loss observed in those taking nintedanib, however this did not reach statistical significance. There was no statistically significant difference in transplant-free survival between patients taking anti-fibrotic medications and those who were not (p = 0.56) (**B**). When patients were stratified by degree of weight loss in the first year from diagnosis, there remained no significant difference based on anti-fibrotic medications (**C**)

To better understand how anti-fibrotic use may impact weight loss on transplant-free survival, we stratified by anti-fibrotic use (Table 4). Patients were assigned to be taking pirfenidone or nintedanib if there were taking that medication following the diagnosis of IPF. There were no significant differences in baseline characteristics of patients taking antifibrotics compared to those not taking antifibrotic medications including average age (71.1 v. 71.9 p = 0.74), DLCO (10.66 v. 12.18, p = 0.17), FVC (2.83 v. 3.01, p = (0.22) or BMI (29.7 v 29.9, p = 0.81). Longitudinal weight changes were then plotted over time. In general, patients on anti-fibrotic medications exhibited similar weight trends as those patients not on anti-fibrotic medications, although there were slight differences in those taking nintedanib compared to patients on pirfenidone or patients not on an anti-fibrotic (Fig. 4A).

**Table 4:** Patient stratification by antifibrotic medications

Characteristic	Nintedanib (n = 19)	PFD (n = 17)	P-value
Age (years)	71.3 ± 10.8	71.0 ± 6.5	0.93
Female Gender	6 (31%)	5 (29%)	0.99
Weight at diagnosis (kg)	87.63 ± 17.2	89.76 ± 15.4	0.70
BMI at diagnosis	30.01 ± 5.2	29.9 ± 4.72	0.94
Smoking (any)	15 (78%)	12 (49%)	0.70
FVC at diagnosis (L)	2.87 ± 0.83	2.79 ± 0.75	0.75
FVC% Predicted	80.0 ± 17.2	89.75 ± 15.4	0.70
DLCO at diagnosis (mL/min/mm Hg)	9.61 ± 3.77	11.78 ± 4.30	0.12
Reflux disease	7 (36%)	7 (41%)	0.99
Diabetes	4	3	0.99
OSA	1	2	0.59
Oxygen use	6	6	0.99

### Weight loss, anti-fibrotic use and mortality

To identify how the use of antifibrotic medications may impact transplant-free survival in our cohort, we performed additional Kaplan–Meier analyses stratifying by use of antifibrotic medications. There was no significant difference in transplant-free survival between anti-fibrotic users and non-users (Fig. 4B). We next wanted to determine whether anti-fibrotic medication use was associated with increased transplant free survival in patients who lost weight as compared to patients who did not lose weight. When stratified by weight loss and anti-fibrotic medication use, the decrease in transplant-free survival was readily apparent in the groups who lost weight (Fig. 4C). However, there was no apparent difference in transplant-free survival on the basis of anti-fibrotic treatment in either the group who lost weight or in those who did not lose weight.

## Discussion

Our study shows that ≥ 5% weight loss over the first year following a diagnosis of IPF is associated with decreased transplant-free survival. The results are consistent with previously reported data, reinforcing the impact of weight loss on mortality. Importantly, our study defines the timing of weight loss as being present immediately after diagnosis rather than occurring later over the disease course. Transplant-free survival in our cohort was 5.35 years but was significantly lower (3.8 years) in patients who lost more than 5% percent of their weight in the first year following the diagnosis of IPF. Critically, transplant-free survival was further decreased (2.02 years) in patients who lost more than 10% of their weight. Thus, we demonstrate an association between severity of weight loss and mortality. Interestingly the median survival of our cohort is 5.35 years which is almost a year longer than what is reported in the literature [26, 27]. It is possible a diagnosis was reached earlier in our patients or possibly by receiving medical care at a Pulmonary Fibrosis Foundation Center. It is also possible that other undocumented lifestyle factors like diet may have contributed to increased median survival in this cohort.

Previous reports on mortality and weight loss in IPF have largely centered around cohorts of patients who were not on an anti-fibrotic medication [15, 18]. However, 52% of the patients in our cohort were taking either pirfenidone or nintedanib. The use of antifibrotic medication is therefore incorporated into the transplant-free survival reported above. Although antifibrotic medications slow the decline in FVC [3–5], when comparing weight loss to FVC in our cohort, unexpected trends emerge. There was no association between weight loss and FVC decline in the first year alone. However, over the entirety of the period of observation, weight loss was associated with FVC decline in a linear manner. This may indicate that weight loss could precede FVC decline in some patients. It may also suggest that weight loss is associated with more aggressive progression of IPF in another subset of patients. Regardless, a > 5% change in weight over the first year after the diagnosis of IPF is an important marker of disease progression and prognosis. Serial weight measurements may therefore have prognostic value in addition to serial FVC measurements, particularly if weight loss occurs in some patients prior to changes in more traditional objective measurements such as FVC.

Our cohort started in 2008, 6 years prior to FDA approval of nintedanib and pirfenidone, and therefore a significant portion of patients did not have these medications as therapeutic options. Weight loss therefore occurs even in the absence of antifibrotic medication use and continues to have a large impact on reducing transplant free survival. Prior reports have shown that use of anti-fibrotic medications is associated with a reduction in mortality. In our cohort, there was no difference in transplant-free survival between patients taking an anti-fibrotic and those who were not (Fig. 4B). Nor was there a difference when mortality analyses were stratified by both weight loss and anti-fibrotic medication. Weight loss appears to have a more significant impact on mortality than any potential benefit from use of antifibrotic medication. However, more research is needed to tease out the mechanisms that surround weight loss in IPF. It is not yet entirely clear whether there may be an improvement in transplant-free survival in patients who do not lose weight and who are taking an antifibrotic medication. Additional studies are required to better define whether which, if either, of the antifibrotic medication contributes to a decrease in transplant-free survival and whether either of the antifibrotic medications improves mortality in patients who do not lose weight.

This study demonstrates the importance of weight loss monitoring immediately after diagnosis in patients with IPF. There are several factors associated with weight loss in chronic disease including nutritional status, and micro- or macro-nutrient intake that were not addressed in this study. The role of proper nutrition in prevention of disease progression or maintenance of the stability is supported by data demonstrating that dietary supplementation in malnourished patients with COPD improved 6-min walking distance (6MWD), respiratory muscle strength (maximal inspiratory and expiratory pressures) and overall health-related quality of life [17]. Furthermore, preclinical data also suggests that specific micro- and macro-nutrient related metabolic pathways may be directly involved the development of pulmonary fibrosis in vivo [28, 29]. Ultimately, additional nutritional epidemiology studies will be critically important to address this knowledge gap.

There are several limitations to our data. As this is a retrospective cohort, patients lacking recorded data were unable to be included. In addition, this is a single center study with a moderate sample size. The presence of diagnosis bias may also be an additional confounder as clinicians have become more adept at recognizing IPF earlier in the course of the disease process. There may be other important confounders that affect an individual’s weight such as diet, nutritional status, concurrent metabolic disorders, amount of physical activity, medication/supplement use, and/or medical co-morbidities that were not clearly documented in each patient’s medical record. In spite of the limitations of the current study, our data support prior observations. The effect of weight loss on mortality in this cohort is striking and certainly warrants additional investigation.

In conclusion, our study shows that ≥ 5% weight loss over the year following a diagnosis of IPF is associated with a significant decrease in transplant-free survival (adjusted HR 2.02). The impact of weight loss on mortality was even more striking in patients who lost more than 10% of their body weight over the first year. Significant weight loss may precede decline in FVC but is independently associated with large volume decline in FVC. Furthermore, there was no apparent increase in transplant-free survival in patients taking antifibrotic medications, but it remains possible that there may be a mortality benefit to anti-fibrotic medications in patients who do not lose weight. Further studies are needed to evaluate the correlation between FVC and weight loss in IPF, as well as to elucidate the underlying mechanism that may put patients with weight loss at risk for IPF progression. Additionally, it remains unknown whether prevention of weight loss by dietary supplementation may mitigate weight loss and the ensuing decrease in transplant-free survival.

## Data Availability

The datasets used and/or analyzed during the current study are available from the corresponding author on reasonable request.
